# Monitoring and quantifying replication fork dynamics with high-throughput methods

**DOI:** 10.1038/s42003-024-06412-1

**Published:** 2024-06-14

**Authors:** Nora Fajri, Nataliya Petryk

**Affiliations:** UMR9019 - CNRS, Intégrité du Génome et Cancers, Université Paris-Saclay, Gustave Roussy, Villejuif, France, 114 rue Edouard Vaillant, 94805 Villejuif, France

**Keywords:** DNA synthesis, Origin firing, Chromatin immunoprecipitation, Histone post-translational modifications, DNA methylation

## Abstract

Before each cell division, eukaryotic cells must replicate their chromosomes to ensure the accurate transmission of genetic information. Chromosome replication involves more than just DNA duplication; it also includes chromatin assembly, inheritance of epigenetic marks, and faithful resumption of all genomic functions after replication. Recent progress in quantitative technologies has revolutionized our understanding of the complexity and dynamics of DNA replication forks at both molecular and genomic scales. Here, we highlight the pivotal role of these novel methods in uncovering the principles and mechanisms of chromosome replication. These technologies have illuminated the regulation of genome replication programs, quantified the impact of DNA replication on genomic mutations and evolutionary processes, and elucidated the mechanisms of replication-coupled chromatin assembly and epigenome maintenance.

## Abstract (web summary)

This review discusses the recent methodological advances in monitoring eukaryotic chromosome replication.

## Introduction

Since the discovery of the DNA double helix structure, the complementarity of two strands immediately suggested an elegantly simple mechanism of DNA replication, where one strand serves as a copy for the other strand^1^. To initiate DNA replication, a pair of replicative helicases unzip the double helix in two directions, creating two DNA replication forks^2,3^. Two genomic strands are oppositely oriented and are replicated differently. The leading strand is copied continuously in the direction of DNA unwinding by DNA polymerase Epsilon (Pol ε)^4,5^. The other arm, the lagging strand, is replicated in short fragments, known as Okazaki fragments, through a more intricate process involving DNA polymerases Alpha and Delta (Pol α and Pol δ)^4,5^. Replication ends when two converging forks meet, generating two daughter duplexes^6,7^.

**Fig. 1:**
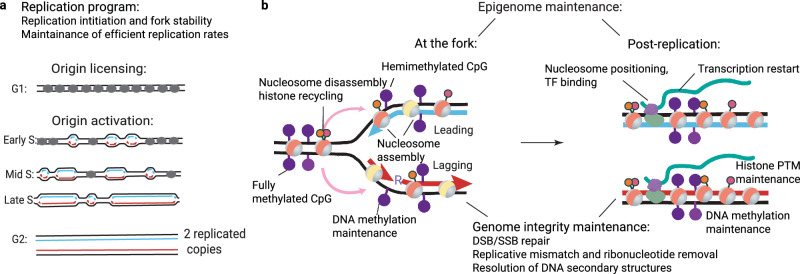
Schematic representation of chromosome replication processes. **a** Temporal regulation of DNA replication at genomic scale. Origins are licensed in the G1 phase (gray) and activated across the S phase (black). Red and blue indicate newly replicated forward and reverse genomic strands. **b** The chromatin replication and epigenome maintenance include replication-coupled (left) and post-replication processes (right). Nucleosome disassembly and assembly at the fork involve recycling of parental modified histones (tangerine) and the new histone deposition (ivory). DNA methylation is maintained after replication, and the newly synthesized strands are remethylated symmetrically to parental strands (violet lollipops). Chromatin maturation and epigenome maintenance entail the transcription restart, re-establishing transcriptional factor occupancy and nucleosome positioning, and progressive post-replicative restoration of histone and DNA modification profiles. Genome integrity processes ensure fork stability and repair of replication mistakes, including excision of incorporated ribonucleotides, mismatches, and nick repair.

For the swift and accurate replication of large eukaryotic genomes, numerous initiation events occur at multiple locations across the genome^8^ (Fig. 1a). Genome replication operates according to the following fundamental principles. First, strict separation of origin licensing (G1 phase) and activation (S phase) prevents re-replication of already replicated regions. Second, DNA replication is chronologically orchestrated across functionally distinct genomic domains and coordinates with transcription and higher-order chromatin organization. Third, the initiation density and fork speeds adapt to attain optimal replication rates and accuracy of DNA replication across the S phase, especially when cells encounter diverse physiological and genotoxic challenges (see reviews^9–12^).

Chromatin structure must be correctly reproduced on newly replicated strands, and genomic functions must resume after DNA replication^13,14^. Chromatin is temporarily disassembled in front of the replication fork and reassembled behind it. Nucleosomes are immediately re-formed on both strands of the fork, requiring twice as many histones as in the parental copy before replication. Conversely, an equivalent amount of new histones, predominantly lacking specific marks, is incorporated in the newly replicated copies^15^. The parental histones carrying modifications are recycled^13^, faithfully maintaining chromatin modification patterns after replication^16^ and ensuring the correct inheritance of chromatin repressive states and genome stability^17–19^. Parental H3-H4 tetramers are distributed to the leading and lagging strands by the coordinated histone chaperone activities of the core DNA replication factors. The DNA polymerase Pol ε subunits, PolE3/4^20^, operate at the leading strand while the histone chaperone activities of replicative helicase subunit MCM2^21^ and DNA polymerase Pol α^22^ operate on the lagging strand. Chromatin assembly factor 1 (CAF1) complements nucleosome density by assembling new H3-H4 tetramers on both daughter strands^23^. Octameric nucleosomes are fully assembled by incorporation of parental and new H2A-H2B dimers shortly after the fork passage^15^. Coordinated regulation between H3-H4 and H2A-H2B histone inheritance pathways has also been reported^24^.

Along with histone marks, DNA methylation of cytosine at CpG sites carries an essential complement of mitotically heritable epigenetic information in many eukaryotes. After replication, DNA molecules are hemimethylated, with methyl groups only present on the original (parental) strands, whereas the newly synthesized strands are initially unmethylated. The enzyme DNMT1, working with the cofactor protein UHRF1, progressively remethylates newly replicated DNA molecules and preserves DNA methylation patterns across cell divisions^25,26^. In summary, the process of chromatin restoration after replication is long and complex. It initiates at the replication fork with the critical steps of histone recycling and nucleosome assembly and continues post-replication. The complete chromatin maturation spans the entire cell cycle, encompassing the resumption of transcription and gradual re-establishment of chromatin structure, restoration of histone modifications, and maintenance of DNA methylation patterns^13,25–27^ (Fig. 1b).

The DNA replication fork is asymmetric and must effectively coordinate the leading and lagging strands. Together with DNA replication, the replisomes must also handle many other tasks, such as DNA damage repair and epigenome maintenance. Genetic assays and biochemical and molecular biology techniques have been instrumental in identifying the critical components of these processes. However, the emergence of quantitative proteomics, high-throughput genomics, and long-read sequencing enables uncovering the complexity and regulation of DNA replication and distinct replication-coupled processes. Many methods for studying DNA replication trace back to the seminal principle of metabolic labeling of replicated DNA^28^. Since then, various approaches have been developed, ranging from microscopic fiber analysis^8,29^ to many more sophisticated readouts (Supplementary Table 1). Moreover, click chemistry, which enables orthogonal labeling and enrichment of replicated DNA^30^, has been instrumental in advancing the field. Over the recent decade, these technologies, tailored to address specific questions, have often yielded surprising and impactful discoveries.

### Measuring replication fork directionality genome-wide

Measuring DNA replication fork dynamics sheds light on fundamental aspects of DNA replication. Replication fork speed and symmetry, and density of replication initiations across the genome are highly responsive to cellular and genotoxic stress^31^. The breakpoints of fork direction across the genome, in other words, changes of fork direction from left to right and from right to left, respectively, reveal DNA replication initiations and terminations. DNA fiber approaches, including DNA combing ^29^, and SMARD^32^, based on two-color labeling of replicated tracks, are powerful direct locus-agnostic methods for measuring fork dynamics that can be performed for individual loci in combination with FISH (Fluorescence In- Situ Hybridization). To reveal the locus-specific dynamics of replication forks at the genomic scale, the proportions of *rightward* and *leftward-oriented* forks, called DNA replication fork directionality (RFD)^33^, can be measured in cell populations by several genomic approaches.

**Fig. 2:**
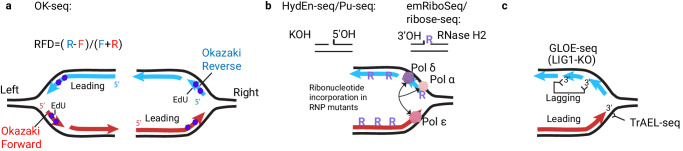
Methods revealing DNA replication fork directionality. **a** Schematic representation of OK-seq: parental DNA strands are black, and newly replicated DNA forward and reverse genomic strands are red and blue, respectively. Okazaki fragment strand reveals fork direction. Reverse Okazaki fragments (blue) originate from the right fork (R), and forward Okazaki fragments (red) originate from the left fork (L). The proportion of red and blue Okazaki read counts reflects the proportion of right and left forks (RFD) across genomic bins. **b** Schematic representation of HydEn-seq/Pu-seq and emRiboSeq/ribose-seq methods. RNR mutants of replicative polymerases leave the embedded ribonucleotides behind the fork (purple). HydEn-seq and Pu-seq use alkaline hydrolysis of the incorporated ribonucleotide to generate 5’-OH and 2’,3’-cyclic phosphate and capture 5’ ends downstream of the initial ribonucleotide incorporation. EmRiboSeq uses enzymatic hydrolysis by RNase H2 to generate 5’-P and 3’-OH ends and to capture the 3’-OH ends upstream of the incorporated ribonucleotide. **c** Schematic representation of GLOE-seq and TrAEL-seq methods detecting 3’-OH ends in a strand-specific manner. GLOE-seq employs DNA denaturation and ligation of double-stranded adapters with random hexanucleotide overhangs using T4 DNA ligase. TrAEL-seq instead uses terminal deoxynucleotidyl transferase (TdT) reaction to add adenosine at 3’ ends followed by single-stranded ligation of a hairpin adapter with T4 RNA ligase. GLOE-seq captures preferentially 3’ OH ends on the lagging stands (blue) in LIG1-depleted cells to reveal the directionality of DNA replication forks. TrAEL-seq detects preferentially the exposed 3’ OH ends on the leading strands (red).

Strand-oriented sequencing and mapping of Okazaki fragments onto forward and reverse genomic strands (OK-seq)^33,34^ allows RFD measurement^33,35^ (Fig. 2a). RFD behavior accurately quantifies the probability of replication fork initiation, progression, and termination in genomes of varying sizes and complexities, including budding yeasts, nematodes, and human cells^33,35–37^. The likelihood of DNA replication initiations is non-uniform across the genome and is influenced by chromatin organization, genome conformation, and transcription^33,37^. Quantifying the absolute number of initiation events per cell is challenging since the patterns vary from cell to cell. However, mathematical modeling and machine learning enable the estimation of local initiation density per cell from population-averaged RFD profiles^38^. Genome-wide approaches must be used hand in hand with the single-molecule methods, which detect the dispersive initiations across the genome, allowing to measure initiation density at the single-molecule level but currently having lower throughput and statistical power compared to short-read-based methods (as discussed below).

Additionally, mapping Okazaki fragment junctions throughout the genome sheds light on the involvement of various factors in the processing of the lagging strand, including the interplay of different nucleases^39^, replication-coupled nucleosome assembly ^34^, and chromatin remodeling^40^. Okazaki fragments can be purified through EdU labeling and click chemistry ^33^, or *LIG1* and checkpoint inactivation^34^. The EdU method captures Okazaki fragments at various stages of maturation and requires a natural or engineered ability to assimilate EdU. Conversely, the depletion of *LIG1*, responsible for Okazaki fragment sealing, captures the mature fragments but alters their original position by strand displacement of unligated nicks^41^.

Replication fork directionality can also be inferred from strand-specific mutational processes, and several methods exploit this principle. Replicative polymerases Pols α, Pol δ, and Pol ε incorporate ribonucleotides into DNA at different rates^42^. The methods detecting strand-specific incorporation of ribonucleotides in DNA polymerase ribonucleotide excision repair (RER) mutants have been developed in several model organisms, including emRiboSeq^43^, HydEn-seq^44^, Ribose-seq^45^ in budding yeasts and Pu-seq^46,47^ in *S. pombe*^46^, and human cells^47^ (Fig. 2b). These methods demonstrate that the leading (Pol ε) and lagging strands (Pols α and δ) share the workload of DNA synthesis, except for some challenging genomic regions where Pol δ plays role in the leading strand replication. These regions include replication origins, termini^46,48^, transcribed genes, and common fragile sites^47^. EmRiboseq reveals that the ribonucleotides incorporated by Pol α during lagging strand replication can persist in the DNA, resulting in genomic mutations^43^. These hotspots often occur near protein binding sites, suggesting chromatin restoration behind the fork can impede the lagging strand processing and ribonucleotide removal^43^. These methods are instrumental for revealing the usage and mutational signatures of the leading and lagging DNA replication polymerases genome-wide but require the design of specific mutations across different model organisms. Strand-specific mutational rates can also be computed from DNA sequences^49^. Distinct leading and lagging strand mutational rates in germline result in strand-specific nucleotide compositional imbalance (skew) accumulation across evolution^50^ and somatic mutations in tumors^51^. While nucleotide composition skew profiles reflect RFD in germline cells^52,53^, their resolution is much lower than that of experimental techniques.

### Methods for revealing strand-specific DNA breaks

Exposure to genotoxic and diverse cellular stresses can result in the generation of DNA breaks. Several groups have developed technologies to map genome-wide DNA double-strand breaks (DSBs). These methods quantify the impact of genotoxic stress on genome integrity and off-target effects of genome editing (reviewed in refs. ^54,55^). BLESS^56^ and BLISS^57^ are based on labeling and capture of DSB ends, while DSBCapture^58^ and END-seq^59^ involve blunting, A-tailing, and direct adapter ligation to the DSB ends. Augmenting the END-seq method with S1 nuclease digestion of single-stranded DNA enables the detection of DNA secondary structures formed in microsatellite repeats during DNA replication and sheds light on the mechanisms driving their instability in cancer cells^60^.

During DNA replication, single-stranded DNA ends transiently emerge along with fork progression and are gradually resolved as replication intermediates mature. Two recent methods, GLOE-seq^61^ and TrAEL-seq^62^ detect strand-specific 3’-OH free ends, enabling the comparison of replication fork dynamics in various experimental conditions (Fig. 2c). Considering the asymmetrical occurrence of 3’-OH at the leading and lagging strands, the ratio between 3’-OH ends mapped to the forward versus reverse strands reveals RFD in wild-type yeast and human cells^61,62^. However, TrAEL-seq shows a stronger RFD signal than GLOE-seq, possibly due to technical differences in library preparation steps^61,62^. Remarkably, both technologies detect 3’-OH ends more frequently in leading strands synthesized continuously than in lagging strands synthesized discontinuously under normal conditions. This observation hints at the high efficiency of Okazaki fragment maturation. Consequently, the Okazaki fragment ligation disruption by *LIG1* mutation inverted and strongly increased the occurrence of 3-OH’ nicks along the lagging strands^61^. In summary, GLOE-seq and TrAEL-seq emerge as powerful tools for monitoring genome integrity during DNA replication.

The methodologies measuring replication fork dynamics genome-wide have significantly enhanced our understanding of DNA replication regulation in large eukaryotic genomes. They offer high-resolution mapping of DNA replication fork initiation, progression, and terminations, providing insights into the mechanisms underlying genome instability and evolution. Furthermore, these techniques have paved the way for discovering mechanisms of post-replicative epigenome maintenance.

### Methods for quantifying replication-coupled chromatin dynamics and post-replicative epigenome maintenance

**Fig. 3:**
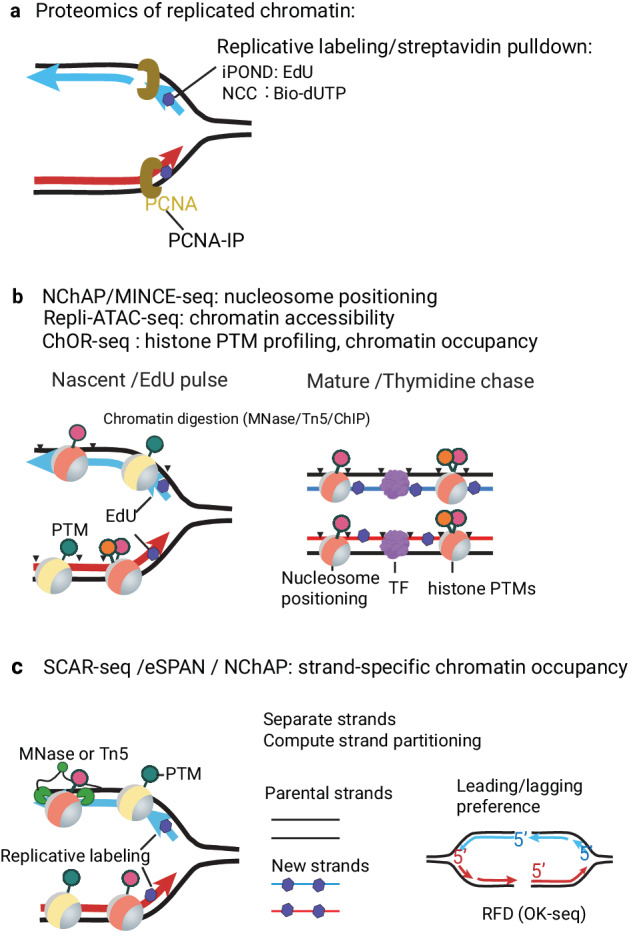
Methods quantifying replication-coupled chromatin dynamics and epigenome maintenance. **a** Proteomic techniques: IPOND, NCC and PCNA-IP. These techniques are based on the purification and proteomic analysis of proteins associated with replicated DNA. IPOND employs labeling of nascent DNA with EdU, followed by biotinylation in click reaction, while NCC employs biotin-dUTP incorporation by hypotonic shock. Sequentially, proteins associated with replicated DNA are enriched with streptavidin pulldown. DNA labeling allows NCC and IPOND techniques to perform pulse-chase experiments and monitor chromatin dynamics post-replication at different time points. Alternatively, proteins associated with DNA replication forks can be purified through PCNA immunoprecipitation. **b** Genomic methods to study chromatin occupancy post-replication: NChAP and MINCE-seq techniques map nucleosome positioning and transcription factors binding sites employing EdU labeling and MNase digestion. Repli-ATAC probes the accessibility of replicated chromatin genome-wide using a Tn5 transposase reaction. ChOR-seq employs sequential ChIP followed by EdU-biotin pulldown to quantify chromatin occupancy of the replicated DNA. In these techniques, nascent time points (EdU pulse) are compared with different time points of thymidine chase (post-replicative chromatin maturation). Exogenous DNA spike-in normalization is used (ChOR-seq) to compare time points quantitatively. **c** Genomic approaches to profile the association of chromatin factors with leading/lagging strands include SCAR-seq, eSPAN, and NChAP. To reveal the strand-specific occupancy, newly labeled strands (red and blue) are isolated from the parental ones and sequenced in a strand-specific manner, and strand partitioning is computed as a ratio of forward and reverse strand counts across genomic bins. The genome-wide association with leading and lagging strand replication is revealed by correlation with RFD, which can be obtained by the OK-seq technique.

The replisomes are highly dynamic multimolecular complexes. DNA replication forks engage with various mechanisms beyond DNA synthesis and chromatin assembly to fully replicate all chromatin components. These mechanisms include epigenome maintenance, DNA repair, sister chromatid cohesion, and higher-order topological organization. Several methods have been developed to reveal the proteome of DNA replication forks in physiological and stress conditions and to elucidate post-replicative chromatin dynamics. Mass spectrometry analysis of proteins associated with labeled DNA, with methods such as iPOND^63^ and the recently improved version iPOND2-DRIPPER^64^, NCC^65^, or co-purification with PCNA^66^ identify new factors related to DNA replication forks and reveals the change in replisome interactors in response to different genotoxic agents and replicative stress^63,64,66–68^ (Fig. 3a). Pulse-chase experiments demonstrate that DNA replication has long-lasting impact on chromatin composition across the cell cycle^13^. The relative abundance of histone marks associated with active and repressive chromatin states oscillates across the cell cycle since the new histones, incorporated during DNA replication, acquire active histone marks faster than repressive marks^69^. Non-histone proteins are transiently dissociated from DNA upon the fork passage and progressively rebind DNA^65,70^. Besides the disruptive impact on chromatin, DNA replication can also promote the binding of some transcriptional factors and chromatin remodellers^70^. Highly informative, mass-spectrometry approaches reveal extensive lists of proteins interacting with DNA replication forks. Developing modeling approaches and deep learning is imperative to exploit these rich datasets and uncover new regulatory pathways^71^. Furthermore, to elucidate the composition of DNA replication forks from functionally distinct genomic regions and chromatin states, the development of locus-specific proteomic essays will be of great importance.

Many genomic technologies have explored the impact of DNA replication on locus-specific chromatin occupancy genome-wide. These include chromatin occupancy profiling with micrococcal nuclease digestion^72–74^, Tn5 transposition^75^, or chromatin immunoprecipitation^16,75,76^, followed by purification of labeled replicated DNA (Fig. 3b). Similarly, several methods have been developed to measure the kinetics of DNA re-methylation post-replication. They employ DNA methylation analysis of replicated DNA by sequencing of bisulfite-treated DNA^77–79^ or  mass-spectrometry^80^, followed by comparison with methylation of parental strands^78–80^ or steady-state methylation^77^. In conjunction with pulse-chase experiments, these diverse chromatin profiling methods elucidate the restoration of distinct chromatin regulatory features and epigenetic components after DNA replication, including nucleosome positioning^72–74^, chromatin accessibility^75^, rebinding of chromatin factors and transcriptional restart after DNA replication^24,75,81^; the kinetics of restoration of histone marks^16,24,76^ and DNA methylation^77–80^. Genomic occupancy methods provide an essential complement to proteomic methods by revealing the locus-specific chromatin dynamics. Their main limitation is that they typically focus on profiling one protein or feature at a time, limiting the comprehension of protein-protein interactions and coregulations.

A significant breakthrough in understanding the mechanisms of replication-coupled epigenome maintenance came with the advancement of strand-specific chromatin profiling of histone marks and chromatin factor associations with the leading and lagging strands of DNA replication forks. These techniques, including NChAP^72^, SCAR-seq^21,76^, and eSPAN^20,82^, rely on the isolation and separation of newly replicated strands from parental strands (Fig. 3c). In budding yeast, the preferential associations with the leading and lagging strands can be revealed by measurement of strand partitioning around known replication origins^20,82^. Genome-wide associations with the leading and lagging strand replication in mammalian cells require quantitative RFD mapping with OK-seq^21,33^. SCAR-seq and eSPAN applied to functional mutants of histone-binding activities of replisome components reveal their role in the inheritance of histone modifications during DNA replication^20–22,24,83^ and post-replicative transposon silencing^84^. In conclusion, these strand-specific genome-wide methods, in combination with mechanistic studies and cryo-electron microscopy (cryo-EM), provide a robust systems approach to understanding chromatin inheritance processes at both the molecular and genomic levels^85^.

### High-throughput single-molecule approaches for profiling replication dynamics

**Fig. 4:**
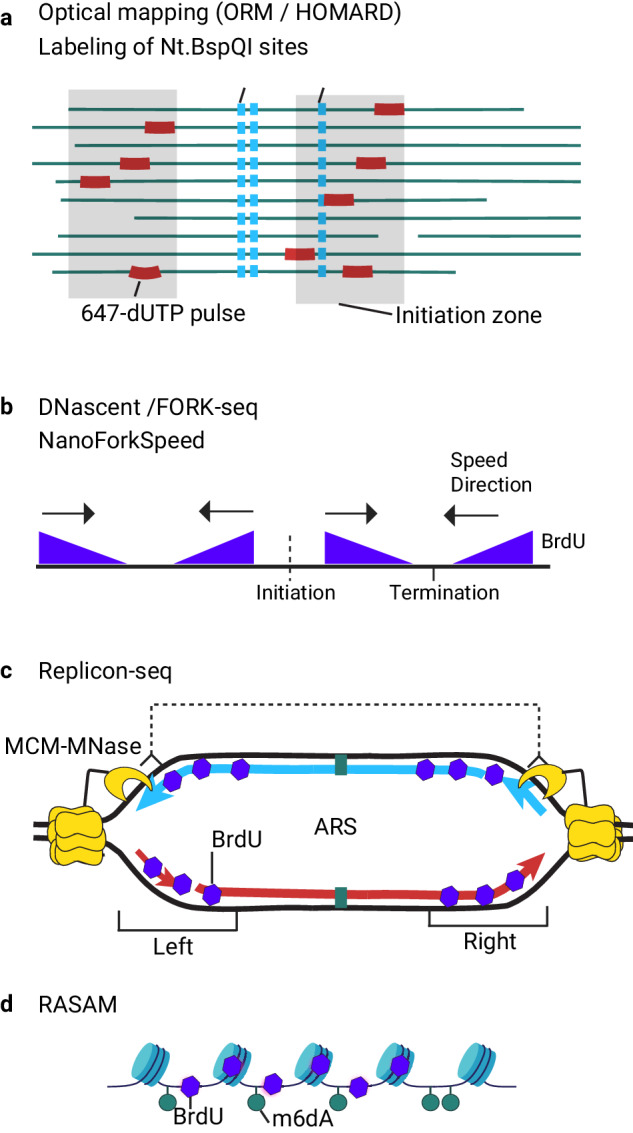
High-throughput single-molecule approaches for profiling replication dynamics. **a** HOMARD and ORM are methods of optical mapping of DNA fibers. DNA replication initiations are detected as incorporation stretches of fluorescently labeled dUTP (red) in DNA replication molecules extracted from cells synchronized in G1/S transition and released into S-phase after electroporation with labeling dUTPs (ORM). HOMARD is optimized for analyzing replicated DNA molecules in vitro assays with *Xenopus* egg extracts. DNA molecules are barcoded and identified through site-specific endonuclease nicking and incorporation of fluorescently labeled dNTPs of a different color (blue). DNA molecules are stained with YoYo-1 (green) imaged with Bionano and aligned using barcoded labels. **b** DNascent, FORK-seq, and NanoForkSpeed (NFS) methods employ replicative labeling detection by nanopore sequencing. Cells are briefly labeled with BrdU, followed by a short thymidine chase. The ongoing rightward and leftward forks are revealed as a decay of BrdU density during thymidine chase (FORK-seq). The length of the BrdU track divided by labeling time provides individual replication fork speed measurement (NanoForkSpeed). Origins and termini are identified as midpoints of diverging and converging replication tracks, respectively. **c** Replicon-seq employs targeted cleavage of intact replicons and analysis by Oxford Nanopore sequencing. For this synchronized yeast cells expressing a fusion of replicative helicase subunit MCM4 with MNase are released in S-phase in the presence of BrdU, followed by targeted cleavage of active replicons by adding calcium, DNA purification, and nanopore sequencing. Replicons are detected as symmetric BrdU stretches and fork symmetry is measured as distance to the ARS within the read. **d** RASAM method. BrdU (purple) is incorporated into nascent DNA, and intact nuclei are incubated with EcoGII m6dA methyltransferase to footprint chromatin accessibility and DNA-protein interactions. Genomic DNA is purified and sequenced using PacBio to detect BrdU and m6dA methylation.

In recent years, new imaging and sequencing platforms have accelerated the development of high-throughput single-molecule technologies to study DNA replication. This includes Bionano DNA molecule imaging^86,87^ and sequencing by Oxford Nanopore^36,88,89^ or PacBio^90^. HOMARD^87^ and ORM^86^ employ DNA fiber imaging where genomic mapping is achieved by an introduction and labeling of site-specific nicks (Fig. 4a). Replicated DNA tracks are detected using the incorporation of fluorescent dUTPs in a cell-free system of *Xenopus* egg extracts^87^ or electroporation in synchronized human cells^86^. These techniques enable high-throughput detection of DNA replication initiations at the single-molecule level in metazoan systems. However, one-color labeling and relatively low resolution do not allow for analysis of single fork dynamics in living cells. The sequencing platforms Oxford Nanopore and PacBio allow the direct analysis of DNA replicative labeling with thymidine analogs. The analogs change the electrical current when the labeled DNA molecule passes through the pore (ONT) and the kinetics of base addition during sequencing (PacBio). Detection models for BrdU, and more recently, for EdU, have been developed by several labs^36,88–91^. Measuring the BrdU incorporation tracks and analyzing the density of BrdU incorporation after pulse-chase labeling, replication fork direction^36,92^, fork speed, and stalling at replication barriers^92^ is detected at the single-molecule level (Fig. 4b). Alternatively, fork direction can be revealed using dual labeling BrdU-EdU in analogy with molecular combing^91,93^. The midpoints between diverging and converging forks map replication initiation and termination events in budding yeasts^36,89^, malaria parasites^93^, and human genomes^94^. Two analog incorporation analyses also allow quantification of replication fork stalling upon replicative stress^91^. Replicon-seq combines BrdU labeling with targeted in situ fork cleavage by MNAse fused with the replicative helicase subunit MCM4 and reveals the dynamics of individual replicons and progression of sister replication forks in budding yeast^88^ (Fig. 4c). Recently, a new technology for single-molecule analysis of chromatin occupancy of replicated DNA molecules using the PacBio platform has been reported. RASAM combines the detection of BrdU labeling and adenine methyltransferase footprinting^90^ (Fig. 4d).

These single-molecule approaches greatly enhance our ability to uncover cell-to-cell heterogeneity of replication fork initiation, progression, and termination. Single-molecule techniques detect initiation events irrespective of their efficiency in cell populations, including dispersive initiations, which are non-visible by population-averaged approaches. However, the limited throughput currently does not allow measurement of initiation and termination efficiency in large metazoan genomes. Optical mapping provides superior coverage depth compared to single-molecule sequencing, whereas the sequencing-based approaches offer superior resolution and exact DNA sequence information. While deepening our understanding of the heterogeneity of the replication process, these novel genome-wide single-molecule methods significantly reduce the methodological and conceptual gaps between single-molecule and high-throughput population-averaged analyses. Averaging single-molecule profiles obtained by FORK-seq of the yeast genome^36^ or DNA combing combined with FISH of two chicken chromosome fragile sites^95^ corroborated the RFD profiling obtained with OK-seq. ORM^86^ and, more recently, DNascent^94^ confirmed that DNA replication initiations in the human genome are dispersive at the single-copy level, occurring more efficiently within initiation zones and less often at termination zones, mapped with cell population methods. With the prospect of increasing throughput, these new single-molecule technologies show promise in becoming the dominant approach for detecting individual replication forks while preserving genomic information.

### Outlook

The advent of high-throughput techniques has revealed previously unreachable aspects of DNA replication dynamics. By integrating these innovative technologies with molecular, biochemical, and structural studies, researchers can craft precise experiments for exploring the intricate connections between various processes crucial for the faithful replication of eukaryotic chromosomes. This includes the interplay between DNA replication, fork stability, and epigenome maintenance. Many core replisome components have conserved dual functions in DNA replication and epigenome maintenance or play a scaffold for recruiting diverse factors, thus mechanistically linking the processes of DNA replication and epigenome maintenance. These novel technologies are pivotal in elucidating these hidden, unconventional roles.

The leading and lagging strand replication mechanisms drive distinct mutational signatures and evolve separate pathways of chromatin assembly and epigenome maintenance. Systematically revealing the similarities and differences between the two strands will illuminate their biological roles and identify potential misregulations in complex diseases.

Proteomic studies have revealed that other than DNA synthesis and repair components, replication forks are enriched with hundreds of proteins with diverse chromatin maintenance and signaling functions, which are not directly related to DNA replication. These factors identified by proteomic studies might be recruited at specific genomic regions to facilitate the smooth navigation of DNA replication forks through diverse chromatin contexts. The replication machinery may be able to adjust its composition locally according to the genomic locus undergoing replication. Novel combinatory approaches are needed to comprehend the locus-specific composition and regulation of DNA replication forks. To achieve this goal, high-throughput genomic and proteomic methods must be employed hand-in-hand with other advanced techniques to determine the structure of replisomes purified from living cells and predict protein-protein interactions in multimolecular complexes.

The advance of long-read sequencing techniques and cell barcoding allows for the heterogeneity of the DNA replication process to be revealed. However, implementing these novel methods also necessitates the development of advanced computational approaches involving machine learning and mathematical modeling. These innovative methods present exciting opportunities to investigate how developmental and metabolic signals, cellular stress, and genomic alterations affect individual replication fork speed, accuracy, and chromatin maintenance processes.

In essence, these novel genomic, proteomic, and computational methods significantly improved our understanding of the critical and fascinating process of chromosome replication. These discoveries hold promise for future applications in biotechnology and precision medicine. For example, pinpointing vulnerabilities in the connections between DNA replication and epigenome maintenance could lead to developing innovative therapeutic approaches for treating cancer.

### Supplementary information

Supplementary Table 1
