# Supplementary material for: Monitoring and quantifying replication fork dynamics with high-throughput methods
Source: Commun Biol. 2024 Jun 14;7:729. doi: 10.1038/s42003-024-06412-1 (PMC11178896; doi:10.1038/s42003-024-06412-1)
Supplement: Supplementary file 1 — Supplementary Table 1 [file 42003_2024_6412_MOESM1_ESM.pdf]

Supplementary Table 1. High-throughput methods for measuring replication fork dynamics.

Name	Abbreviation	Key features
**High-throughput methods to study DNA replication fork dynamics**		
Okazaki fragment sequencing	**OK-seq**[33,34]	- Measures replication fork directionality by isolating and mapping of Okazaki fragments<br>-Reveals locus-specific probability of replication initiation and termination.<br>- Reveals the dynamics of Okazaki fragment processing and replication-coupled nucleosome assembly.<br>- Okazaki fragments can be enriched through EdU incorporation or *LIG1* inactivation.<br>- Applied in yeast and cell lines from different vertebrate species, including primary human cells and *C. elegans* embryos.
Embedded Ribonucleotide Sequencing<br>Polymerase usage sequencing<br><br>Hydrolytic end sequencing	**emRiboSeq**[43]<br>**Ribose-seq** [45]<br>**Pu-seq**[46,47]<br>**HydEn-seq**[44]	- These methods detect ribonucleotide incorporation and replicative polymerase usage during DNA replication.<br>- Reveal mutational signatures of leading and lagging DNA replication polymerases genome-wide.<br>- Can be used to measure RFD and high-resolution profiling of replication origins and termini.<br>- Require construction of specific RNR-deficient mutants.
Genome-wide ligation of 3′-OH ends sequencing.<br>Transferase-Activated End Ligation sequencing.	**GLOE-seq**[61]<br><br>**TrAEL-seq** [62]	- Detect 3'-OH free ends of SSBs and DSBs in a strand-specific manner.<br>- Reveal the distribution and frequency of 3' ends allowing to detect replication-associated DNA damage and measure RFD.
**High-throughput methods to study chromatin assembly and epigenome maintenance**		
Chromatin occupancy after DNA replication	**ChOR-seq**[16]	- Profiles the abundance of protein/histone modifications by sequential chromatin precipitation, purification and sequencing of new labeled DNA with EdU.<br>- Reveals the distribution of histone modifications on newly synthesized DNA strands and measures the restauration of chromatin occupancy and histone PTMs in pulse-chase experiments.
Mapping in vivo nascent chromatin with EdU and sequencing<br><br>Affinity purification of EdU containing nucleosomal DNA [74]	**MINCE-seq**[73]	- Maps nucleosome positioning in replicated DNA by MNAse digestion.<br>- Provides nucleosome occupancy dynamics post-replication in pulse-chase experiments.
Replication-coupled assay for transposase-accessible chromatin	**Repli-ATAC-seq**[75]	- Profiles chromatin accessibility genome-wide post-replication with Tn5 transposase and isolation of new labeled DNA with EdU.

		- Predicts TF factor occupancy post-replication and measures the chromatin accessibility restoration post-replication in pulse-chase experiments.
Nascent chromatin avidin pulldown	**NChAP**[72]	- Maps nucleosome positioning in replicated DNA using MNase digestion and isolation of newly synthesized DNA labeled with EdU.<br>- Detects nucleosome positioning on leading and lagging strands.
Enrichment and sequencing of protein-associated nascent DNA	**eSPAN**[20,82]	- Maps chromatin occupancy and distinguishes between two newly synthesized DNA strands.<br>- Employs purification of newly synthesized DNA labeled with BrdU after ChIP or CUT&Tag chromatin profiling.<br>- Requires measurement of replication directionality to assign leading and lagging strands (or relies on position of replication origins in yeast)<br>- Applied in functional mutants reveals the role of replisome components in replication-coupled epigenome maintenance.
Sister chromatids after replication	**SCAR-seq**[21]	- Maps the relative abundance of histone PTMs and chromatin-bound factors on the newly replicated DNA strands in a strand-specific manner.<br>- Employs purification of newly-synthesized DNA labeled with EdU after native or xChIP.<br>- Maps chromatin occupancy and distinguishes between two newly synthesized DNA strands.<br>- Allows independent strand-specific analysis of parental strands.<br>- Employs genome-wide correlative analysis with RFD to reveal associations of protein occupancy with the leading and lagging strands.<br>- Applied in functional mutants reveals the role of replisome components in replication-coupled epigenome maintenance.
**High-throughput methods for quantifying post-replicative DNA methylation maintenance**		
Bisulfite sequencing of replicated DNA	**Repli-BS**[77]	- Bisulfite treatment of BrdU-labeled purified replicated DNA.<br>- Employs bisulfite analysis pipelines; compares DNA methylation levels between nascent and bulk bisulfite treated samples.
Hairpin-assisted mapping of methylation of replicated DNA	**Hammer-seq**[79]	- Sequential EdU pull-down and bisulfite treatment of replicated DNA.<br>- Hairpin ligation maintains parental and new stands in same read.<br>- Parental and new strands are assigned to strands with higher and lower methylation levels in pair respectively.
Nascent DNA Bisulfite Sequencing/ Chromatin Immunoprecipitation on Nascent Chromatin followed by bisulfite sequencing	**nasBS-seq/nasChIP-BS-seq**[78]	- DNA methylation analysis of replicated DNA and chromatin occupancy of methylated and hemi-methylated DNA.<br>- Employs sequential EdU pull-down, purification, parental and new strand separation, bisulfite treatment of EdU-labeled DNA and strand-specific sequencing.

		-Sophisticated analysis pipeline and in silico model for pairing of parental and new strands to reveal DNA methylation maintenance in CpG dyads.
**High-throughput single-molecule techniques to study DNA replication and replicated chromatin**		
Optical replication mapping / High-throughput method for optical mapping of replicated DNA	**ORM**[86]<br><br>**HOMARD**[87]	- Replicative incorporation of fluorescent d-UTP requires cell electroporation or in vitro assays.<br>- High-throughput single-molecule methods applicable in vertebrate systems.<br>- Does not allow to analyze individual replication forks<br>- Mean fiber length is relatively short as compared to DNA combing and does not allow inter-origin distance analysis
Analysis of nascent DNA by nanopore sequencing	**DNAscent[89]/ FORK-seq[36] / NanoForkSpeed[92]**	- Nanopore sequencing to detect individual replication forks by BrdU incorporation.<br>- Detect replication initiations irrespectively of their efficiency in cell population.<br>- The throughput is currently limited and application in vertebrate systems and requires development of enrichment strategies.<br>- Mean read length is relatively short as compared to DNA combing and does not allow inter-origin distance analysis.
Detection of DNA replication forks using nanopore sequencing	**Replicon-seq[88]**	- BrdU labeling followed by targeted cleavage of DNA by MCM4-MNAse fusion protein.<br>- Measures speed and symmetry of two forks of within a single replicon in budding yeasts.<br>- Controlled yeast cell synchronization and release allow to follow replicons at different stage of replication progression.<br>- Requires engineered yeast strains able to assimilate BrdU and carrying fusion MCM4-MNAse protein. These mutations may compromise DNA replication dynamics.
Replication-Aware Single-molecule Accessibility Mapping	**RASAM**[90]	- Maps chromatin occupancy post-replication, by adenine methyltransferase footprinting of DNA accessibility and BrdU detection using PacBio sequencing.<br>- Allows single-molecule analysis of restoration of post-replicative chromatin accessibility and occupancy.<br>- The throughput is currently limited, locus-specific analysis of large genomes requires development of enrichment strategies.
